# ^18^F-FDG PET biomarker of individual cerebral metabolic connectivity in focal epilepsy subtypes and association with prognosis

**DOI:** 10.3389/fmed.2026.1817841

**Published:** 2026-06-16

**Authors:** Li Li Liu, Zhen Peng Chen, Zhehao Lyu, Dongxue Wang, Bing Han, Ping Li

**Affiliations:** 1 The Second Affiliated Hospital of Harbin Medical University, Harbin, China; 2 Shandong University of Traditional Chinese Medicine Qingdao Academy of Chinese Medical Sciences, Qingdao, China; 3 First Affiliated Hospital of Harbin Medical University, Harbin, China

**Keywords:** ^18^F-FDG PET, cerebral, focal epilepsy, individual, metabolic connectivity, prognosis

## Abstract

**Purpose:**

This research aimed to comprehensively explore the divergent topological alterations in metabolic connectivity associated with focal epilepsy, including right temporal lobe epilepsy (RTLE), left temporal lobe epilepsy (LTLE), extra temporal lobe epilepsy (ETLE), and their associations with prognosis.

**Methods:**

This study enrolled a cohort of patients who had been diagnosed with focal epilepsy, including RTLE, LTLE, and ETLE. The metabolic connectivity of each individual, determined by the first-order features of ^18^F-FDG PET SUV parameters, was used to conduct a comprehensive analysis of the cerebral metabolic network. Alterations in network connectivity were assessed by extracting and evaluating the strength of edges, node connectivity, and their associations with prognosis.

**Results:**

Compared with the health controls (HCs), the RTLE, LTLE, and ETLE groups exhibited substantial alterations in metabolic network connectivity at the edge and node levels (*p* < 0.01, FDR-corrected). In RTLE patients, the key disparity nodes were the right Rolandic area, bilateral cerebellum, and left superior parietal gyrus. In LTLE, the notable variations were the left middle temporal gyrus, the right fusiform gyrus, the left thalamus, and the right calcarine area. In ETLE patients, the network abnormalities were related to the unilateral postcentral gyrus, bilateral supramarginal gyrus and putamen, precentral gyrus, right thalamus, and caudate. Regarding prognosis, in the ETLE group, edge connectivity of some cerebral regions showed a positive correlation with the prognosis classifications.

**Conclusion:**

The research demonstrates distinct topological characteristics of metabolic networks in focal epilepsy, which may provide a foundation for understanding clinical symptomatology and inform future investigations into imaging-based biomarkers.

## Introduction

Epilepsy is a chronic, non-communicable brain disease that affects individuals of all ages and represents one of the most commonly diagnosed neurological conditions worldwide, with an estimated prevalence of more than 50 million individuals and an annual incidence of two million (1). Focal epilepsy is the most common type (2), caused by hippocampal sclerosis (HS), tumors, focal cortical dysplasia (FCD), hemorrhage, or other structural lesions. Furthermore, depending on the location of the lesion, temporal lobe epilepsy (TLE) is the most common type, followed by frontal lobe epilepsy (FLE), which accounts for 20–30% of all focal epilepsy (3, 4). Recent studies have revealed that both TLE and extra-temporal lobe epilepsy (ETLE) are characterized not only by localized lesions in the epileptic zone (EZ) but also by disruptions in the global brain network (5, 6).

2-Deoxy-2-[^18^F]fluorodeoxyglucose positron emission tomography (^18^F-FDG PET) is routinely performed in preoperative evaluations of focal epilepsy in patients using magnetic resonance imaging (MRI)-negative or inconclusive findings. The analysis serves as an informative tool for identifying potential EZs in conjunction with other findings (7), and may provide data in terms of signal-to-noise ratios, out-of-sample replication, and variance concentration than functional MRI (fMRI) data (8, 9). Additionally, ^18^F-FDG PET can be used to construct a metabolic connectivity network that directly reflects neuronal energy demands. To date, ^18^F-FDG PET has been used to explore metabolic connectivity in healthy controls (HCs) (10) and in individuals with various neuropsychiatric disorders, including autism (11), Alzheimer’s dementia (12), and Parkinson’s disease (13). Moreover, the variances in the metabolic network between mesial TLE (MTLE) and neocortical TLE (NTLE) (14) were also explored. Several studies have demonstrated that extra-EZ hypometabolism is a consequence of prolonged seizure attacks and reflects the extension of the epileptic network (15). Previous studies have revealed disrupted metabolic networks, increased network integration, and metabolic connectivity in individuals with TLE experiencing ongoing postoperative seizures. This study focused on lateralized TLE (Left TLE and right TLE) and ETLE, aimed to investigate hemisphere-specific and lobe-specific network alterations, rather than distinguishing the mesial and neocortical subtypes within the temporal lobe.

However, limited number of studies have explored ^18^F-FDG PET parameters for constructing the whole brain metabolic connectivity in individual patients. The objective of this study was to investigate the changes in metabolic connectivity in RTLE, LTLE, and ETLE patients compared with HCs and to explore the differences in clinical characteristics and the factors associated with prognosis.

## Materials and methods

### Patients and healthy controls

A total of 116 patients with focal epilepsy (including 45 patients with LTLE, 44 patients with RTLE, and 27 patients with ETLE) from the Second Affiliated Hospital of Harbin Medical University between October 2017 and December 2025 were retrospectively reviewed. According to the International League Against Epilepsy (ILAE) criteria, the clinical diagnoses were established based on comprehensive clinical evaluations (2, 16).

**Figure 1:**
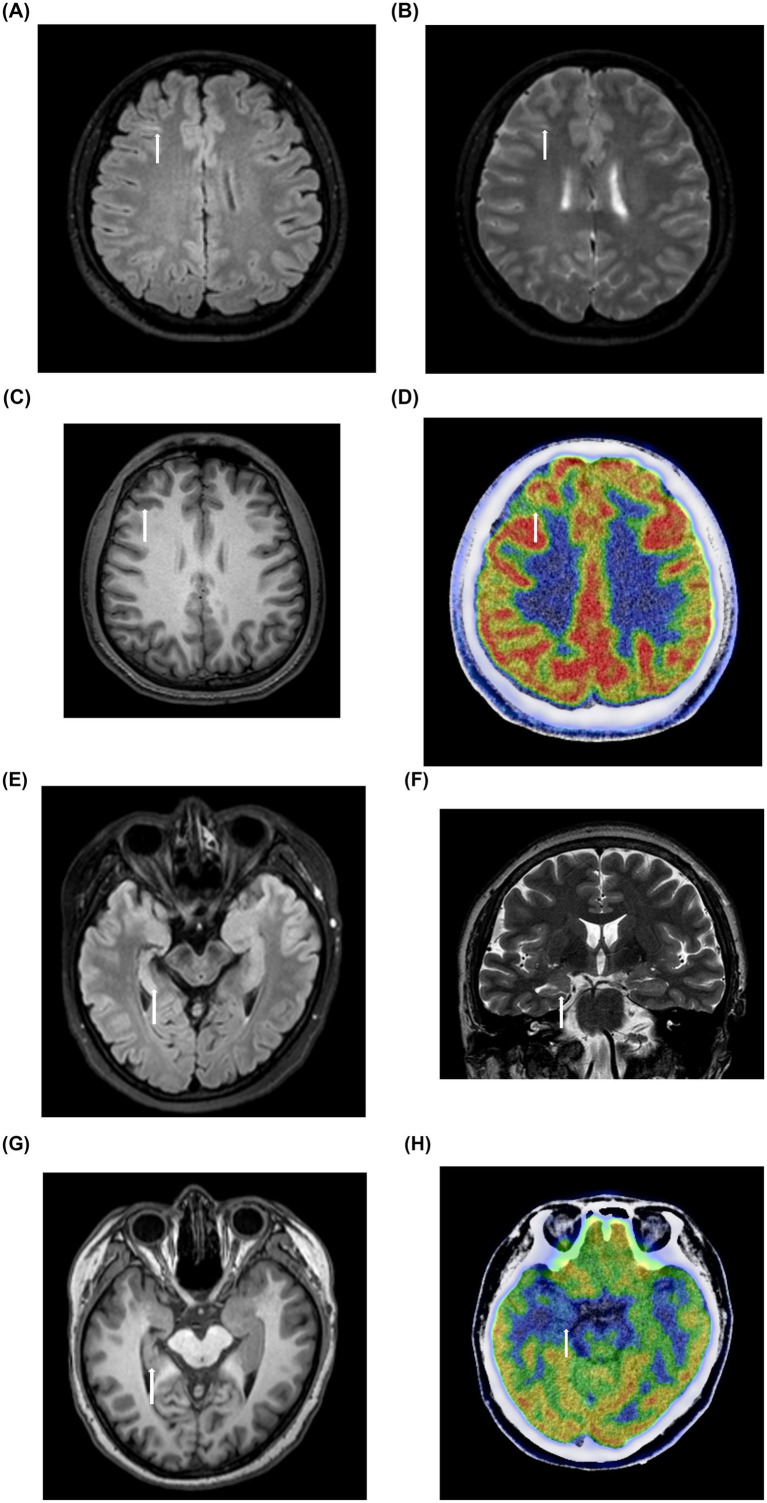
Case 1. FCDII type patient, accompanied by epilepsy for 5 years, with positive MR and PET manifestations. **(A–C)** Right middle frontal gyrus in axial and coronary FLAIR, T2WI, and T1WI in MRI showed abnormal signals (thick white arrow). **(D)** Hypometabolism of the right middle frontal gyrus in ^18^F-FDG PET (thick white arrow). Case 2. Right hippocampus sclerosis patient, with epilepsy for 2 years, **(E–G)** showed bilateral hippocampus in axial FLAIR, T2WI, and T1WI in MRI, showing abnormal signals. Right hippocampus was atrophy, and the signal was higher (thick white arrow). **(H)** Hypometabolism of the right hippocampus in ^18^F-FDG PET (thick white arrow).

Among the 116 enrolled patients, none presented a prior history of neurological disorders apart from epilepsy. Detailed clinical information was collected for each participant, encompassing seizure semiology, duration, frequency, and familial history. All subjects completed a standardized neuroimaging evaluation consisting of video-electroencephalography (VEEG), 3.0-Tesla brain MRI, and ^18^F-FDG PET/CT. The lateralization and localization of EZ and treatment strategy were determined by a multidisciplinary team (MDT) consisting of five experienced neuroelectrophysiologists and neurophysicians, two neuroradiologists, and one neurosurgeon. All patients received regular antiepileptic drug (AED) treatment. For standardized assessment of prognosis, the ILAE outcome classification serves as a common framework for both patients after epilepsy surgery and those managed with AEDs, enabling comparative outcome evaluation (17). Forty-five HCs matched for age and gender were enrolled, with exclusion criteria including other neurological and psychiatric diseases, and drug addiction. Representative MRI and ^18^F-FDG PET images from two patients included in this study—one with FCD and one with HS, illustrate the characteristic structural and metabolic abnormalities observed in the study cohort (Figure 1).

### MRI acquisition

The MRI scanner utilized was a 3.0 Tesla Discovery 750w instrument (GE Healthcare, United States). The standard MRI protocols included T1-weighted imaging (T1WI), fluid-attenuated inversion recovery (FLAIR), T2-weighted imaging (T2WI), and diffusion-weighted imaging (DWI). For T1WI [repetition time(TR) = 701 ms, echo time (TE) = 3.17 ms, and field of view(FOV) = 240 mm × 240 mm], T2WI (TR = 10,000 ms, TE = 81 ms, and FOV = 200 mm × 200 mm), FLAIR (TR = 5,000 ms, TE = 341 ms, and FOV = 220 mm × 220 mm), and DWI (TR = 2,500 ms, TE = 87.67 ms, and FOV = 200 mm × 200 mm), axial, coronary, and sagittal images were obtained, and the T1WI, FLAIR, and T2WI slice thicknesses were 1 mm, 1 mm, and 2 mm, respectively. Two experienced radiologists independently evaluated the MR images, and an informed consensus was reached.

### PET image acquisition

The PET/CT scanner utilized a Siemens Biograph 64 time-of-flight instrument. All the patients were discontinued from AEDs for at least 24 h and asked to fast for at least 6 h before imaging. The patients were required to rest quietly in a dedicated room to ensure minimal auditory and visual stimulation after injection. Moreover, fasting blood glucose levels could not exceed 8 mmol/L. The dose of imaging agent (^18^F-FDG) injected was 4.44 MBq/kg. The brain acquisition time was 10 min. PET images were reconstructed using a TrueX algorithm (5 iterations, 21 subsets) with time-of-flight (TOF) information. The reconstructed image matrix was 400 × 400, resulting in a voxel size of 1.0 × 1.0 × 1.0 mm^3^. A 2 mm full-width at half-maximum (FWHM) and Gaussian post-filter were applied.

### Scenium software methods in ^18^F-FDG PET imaging analysis

Scenium software (Version VD10, Siemens Medical Solutions USA, Inc) provides quantification tools for the assessment of ^18^F-FDG-PET images to perform a color-coded statistical analysis and highlight patterns of unusual radiopharmaceutical uptake. This software uses a deformable fusion algorithm to fuse the patient images with a healthy control database. Region of interests (ROIs) were obtained under license from the Commissariat à l’Énergie Atomique et aux Énergies Alternatives (CEA) or Groupe d’Imagerie Fonctionnelle (18). The cerebral regions were divided into 94 subregions (excluding the brainstem) according to the automated anatomical labeling (AAL) standards.

For each AAL-defined brain region, the following parameters were extracted: (1) SUV_mean_: the mean standardized uptake value within the ROI, (2) SUV_meanstd_: the standard deviation of SUV_mean_ values within the ROI, (3) SUV_max_: the maximum voxel SUV value within the ROI, and (4) SUV_maxstd_: the standard deviation of SUV_max_ values within the ROI. SUV values were normalized by body weight (BW), calculated as,


$$\mathrm{SUV}=\frac{\text{Tissue Activity Concentration}\;(\mathrm{kBq}/\mathrm{mL})\;}{\left[\text{Injected Dose}\;(\mathrm{kBq})/\text{Body Weight}\;(g)\right]}$$

The cerebellum was selected as the reference region because it is less affected by partial volume effects than the pons and exhibits relatively preserved metabolism in unilateral TLE compared to the global cortical mean, which is often significantly depressed ipsilaterally. Furthermore, analysis of the asymmetry index in the study cohort showed no significant interhemispheric cerebellar metabolic asymmetry (AI <0.05) across all groups. The SUVR was calculated using the cerebellum as the reference region (19), according to the following formula:


$$\text{SUVR}=\frac{\mathrm{SUV}(\mathrm{ROI})}{\mathrm{SUV}(\text{cerebellum})}$$

The ^18^F-FDG PET images were analyzed through the Scenium software, performed by drawing the ROIs and calculating both the SUVs and SUVR.

### ^18^F-FDG PET brain metabolic network construction

The first-order features of ^18^F-FDG PET parameters included SUV_mean_, SUV_meanstd_, SUV_max_, SUV_maxstd_, and SUVR. The different statistic-based features based on SUVs.

First, this study adopted the *Z* score normalization method to unite the feature scale, which can be formulated as:


$$z=\frac{x–\mu }{\sigma }$$

Where μ and σ denote the mean and the standard deviation values, respectively.

Second, correlation coefficients across subjects between each pair of brain regions were calculated separately for the case and control groups. To construct individual-level metabolic networks, this study adopted a feature-based similarity approach. For each subject, a vector comprising eight first-order statistical features (SUV_mean_, SUV_meanstd_, SUV_max_, SUV_maxstd,_ and their corresponding SUVR) was extracted from each of the 94 ROIs. This approach, inspired by prior work on morphometric similarity networks (20), emphasized that extracting multiple morphological features from each region to form a vector and calculating the similarity as the connective value (21) assumes that regions with similar uptake statistical distributions exhibited higher metabolic connectivity. This relationship can be formulated as:


$$R_{\mathrm{XY}}=\frac{\sum_{i=1}^{n}(X_{i}-\bar{X})(Y_{i}-\bar{Y})}{\sqrt{\sum_{i=1}^{n}{\left(X_{i}-\bar{X}\right)}^{2}}\sqrt{\sum_{i=1}^{n}{\left(Y_{i}-\bar{Y}\right)}^{2}}}$$

Where X and Y denote the statistic-based features extracted from brain regions X and Y , respectively, and n represents the number of the first orders, individual brain metabolic networks of size 94 *94 according to the AAL atlas. These networks were constructed to represent interregional similarity connections derived from the statistical features.

Third, to improve the normality for subsequent statistical comparisons, the correlation coefficients were transformed using the Fisher-*Z* transformation:


$$Z_{r}=\frac{1}{2}\ln(\frac{1+R_{\mathrm{XY}}}{1-R_{\mathrm{XY}}})$$

### Visualization of metabolic connectivity matrices

This study further interpreted the most discriminative connections between ROIs based on edge importance probability. Two-sample *t-*tests were conducted on the Brain Connectivity Matrix Heatmap to detect the most discriminative connections between three subtypes of focal epilepsy (including RLTE, LTLE, and ETLE) vs. HCs. The top 25 most discriminative connections (false discovery rate, FDR; corrected *p* < 0.01) between brain ROIs in the RTLE group vs. HCs were listed. In addition, the top 25 most discriminative connections using the same standard, for LTLE vs. HCs and ETLE vs. HCs, were listed.

### Standard protocol approvals, registrations, and patient consent

This study received ethical approval from the Institutional Review Board of the Second Affiliated Hospital of Harbin Medical University (number KY2023-170), and written informed consent was obtained from all the enrolled patients.

### Statistical analyses

Statistical analyses were performed using SPSS 25.0 (IBM Corp) and GraphPad Prism 9.4.1 (GraphPad Software, United States). Categorical variables were compared and analyzed using the Fisher’s exact test. The data are presented as the mean ± SDs for continuous variables with a normal distribution, and the data for non-normally distributed variables are expressed as the medians [interquartile ranges (IQRs)]. Continuous variables were analyzed using parametric (Student’s *t*-test) or non-parametric (the Mann–Whitney *U-*test) tests for two-group comparisons. For multigroup comparisons, the analysis of variance (ANOVA) or the Kruskal–Wallis test was used.

For the edge-wise comparisons between each patient subgroup and the HC group, two-sample *t*-tests were performed on the Fisher *Z*-transformed correlation coefficients. To control for multiple comparisons within each subgroup analysis, the FDR correction was applied across all the edges simultaneously using the Benjamini–Hochberg procedure. An adjusted *p-*value threshold of <0.01 was considered statistically significant.

Furthermore, given the ordinal nature of the ILAE outcome classification (graded I–VI), the exploratory Spearman’s rank correlation analyses were conducted to investigate potential monotonic relationships between prognosis and individual network topological attributes. It is acknowledged that the ordinal logistic regression may represent a more statistically rigorous approach for modeling the full distribution of ordinal outcomes; however, the limited sample size within each outcome category precluded stable model fitting in the present cohort. Accordingly, these correlation analyses were presented as hypothesis-generating observations and interpreted with appropriate caution. Statistical significance for exploratory analyses was set at a *p* < 0.05.

## Results

### Part 1. Clinical results

Autonomic symptoms are seen commonly in TLE patients (35/54, 39.3%) than the ETLE patients (5/22, 18.5%; *p* = 0.002; Table 1). Duration in focal epilepsy was longer in the TLE group than the ETLE group (*p* = 0.117). There was no difference among the three groups in epilepsy during sleep (*p* = 0.417). In Table 2, the proportion of male patients is significantly higher in frontal lobe epilepsy compared with female patients. Seizure duration was significantly longer in patients with parietal lobe epilepsy than in those with frontal lobe epilepsy. No significant intergroup differences in autonomic nervous system symptoms were found among the four ETLE subgroups.

**Table 1:** Demographics and clinical characteristics of focal epilepsy patients, including RTLE, LTLE, ETLE, and controls.

Clinical information	Controls (*n* = 45)	RTLE (*n* = 44)	LTLE (*n* = 45)	ETLE (*n* = 27)	*P-*value
Sex, M/F, *n*	25/20	25/19	29/16	17/10	0.744^*^
Age at the time of the study, years, mean ± SD	38.32 ± 10.92	33.25 ± 13.65	34.51 ± 12.68	32.30 ± 14.61	0.701^#^
Duration, years, mean ± SD		10.52 ± 11.09	13.18 ± 10.78	8.22 ± 7.30	0.117^#^
Aura manifestation (yes/no)		23/21	19/26	8/19	0.172^*^
		42/47		8/19	0.107^*^
Epilepsy during sleep (yes/no)		34/55		8/19	0.417^*^
Autonomic symptoms (yes/no)		24/20	11/34	5/22	0.002^*^
Years to last follow-up, years, mean ± SD		1.77 ± 1.38	1.52 ± 1.13	1.46 ± 1.14	0.803^#^
Results of MRI (positive/negative)		29/15	31/14	23/4	0.191^*^
		60/29		23/4	0.073^*^
Treatment outcome (good/bad)		25/19	27/18	20/7	0.325^*^
		52/37		20/7	0.142^*^
Histopathology, *n* (%)					
HS		5(14.7%)	3(8.8%)		
Gliosis			1(2.9%)	10(29.4%)	
FCD		4(11.8%)	1(2.9%)	7(20.5%)	
Cavernous angioma		3(8.8%)			

RTLE, right temporal lobe epilepsy; LTLE, left temporal lobe epilepsy; ETLE, extra-temporal lobe epilepsy.

ETLE including frontal and parietal epilepsy, the former was 17 cases, the later was 10 cases.

# Kruskal–Wallis test, *chi-square test (including Fisher’s exact test).

**Table 2:** Demographics and clinical characteristics of ETLE patients.

Clinical information	Right frontal epilepsy (*n* = 12)	Left frontal epilepsy (*n* = 5)	Right parietal epilepsy (*n* = 5)	Left parietal epilepsy (*n* = 5)	*p*-value
Gender, M/F, *n*	9/3	5/0	2/3	1/4	0.029^*^
Age at the time of the study, years, mean ± SD	38.07 ± 14.35	41.84 ± 17.99	43.50 ± 8.87	25.95 ± 6.38	<0.001^#^
Duration, years, mean ± SD	11.23 ± 6.11	9.85 ± 4.90	14.86 ± 7.86	20.13 ± 8.68	<0.001^#^
Aura manifestation (yes/no)	3/9	1/4	1/4	3/2	<0.001^*^
Epilepsy during sleep (yes/no)	5/7	1/4	0/6	2/3	0.281^*^
Autonomic symptoms (yes/no)	2/10	0/5	2/3	1/4	0.582^*^
Results of MRI (positive/negative)	9/3	5/0	5/0	5/0	0.311^*^
Treatment outcome (good/bad)	9/3	1/4	2/3	5/0	0.029^*^

# Kruskal–Wallis test, *chi-square test (including Fisher’s exact test).

### Part 2. Metabolic network topology in different groups

**Figure 2:**
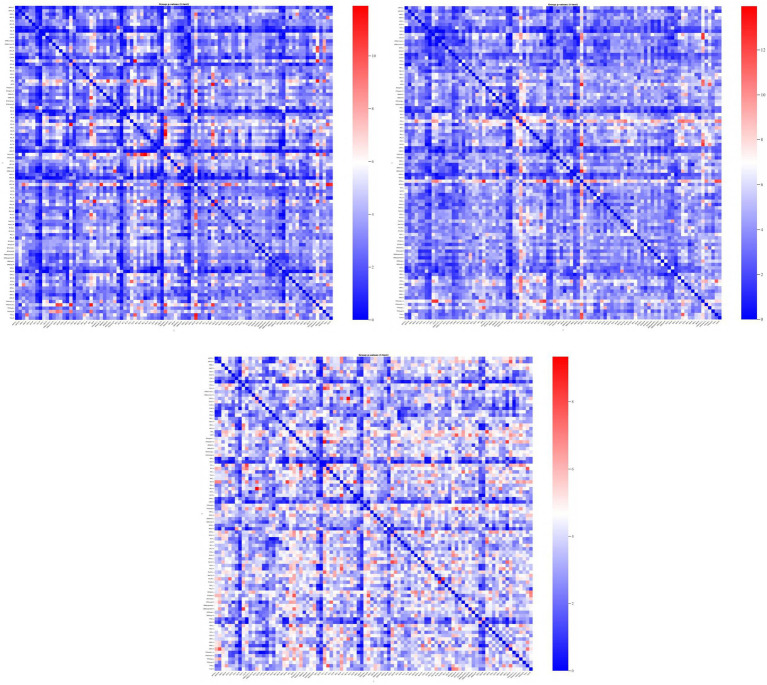
**(A)** Heatmap of the metabolic connectivity topology between the RTLE group and HCs according to the AAL regions. This heatmap illustrates the inter-group comparison of correlation coefficients between brain regions, derived from first-order feature matrices. For each individual, a 94 × 94 correlation matrix was constructed using computing pairwise correlation coefficients between every two ROIs. The same procedure was applied to the normal control group. Subsequently, inter-group comparisons were performed, yielding eight *p*-value matrices corresponding to the eight first-order features. Each sub-matrix in the heatmap represents the comparison between two ROIs relative to the normal control group. In the color scheme, blue indicates a lower correlation coefficient with a larger *p*-value, whereas red represents a higher correlation coefficient with a smaller *p*-value. **(B)** Heatmap of the metabolic connectivity topology between the LTLE group and HCs according to the AAL regions. **(C)** Heatmap of the metabolic connectivity topology between the ETLE group and HCs according to the AAL regions.

**Figure 3:**
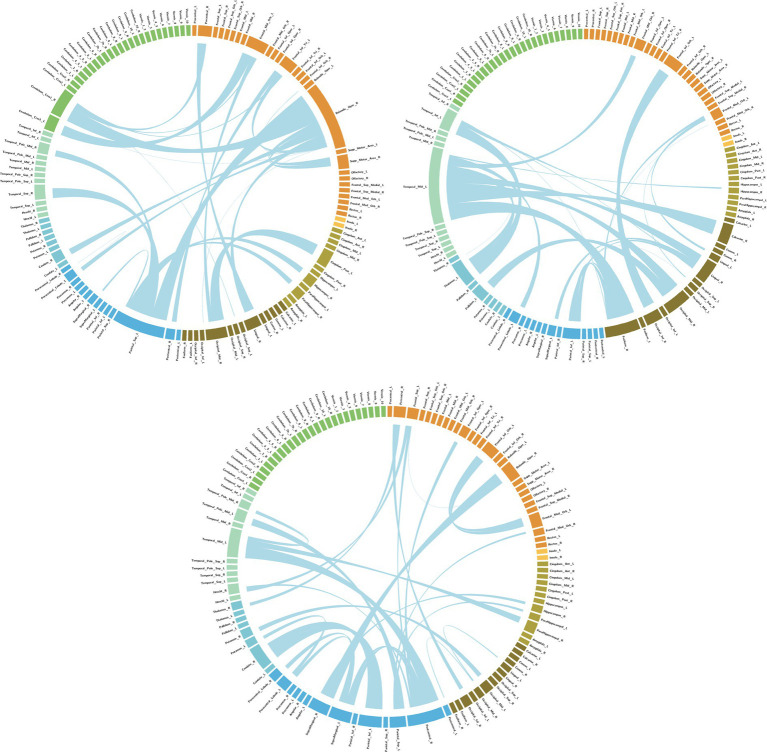
Top 25 metabolic connections between brain regions were identified by the greatest correlation coefficients. **(A)** In the RTLE group, right Rolandic area to left superior parietal gyrus, right supplementary motor area, right caudate nucleus, right cerebellar crus1, right lingual gyrus, left paracentral lobe, left inferior frontal gyrus, triangular part, left Rolandic area, left inferior occipital gyrus, and left cerebellar crus 1; right middle occipital gyrus to left posterior cingulate gyrus; left middle orbital gyrus to left cerebellar crus 1; left superior parietal gyrus to right superior temporal gyrus, right middle temporo-parieto-occipital junction, right hippocampus, right parahippocampal gyrus, and right supramarginal gyrus; right lingual gyrus to right precentral gyrus; left inferior frontal gyrus and triangular part to right postcentral gyrus; right middle occipital gyrus to left middle orbital gyrus; right cerebellar Crus 1 to right precentral gyrus, left middle orbital gyrus, left inferior frontal gyrus, triangular part, and right superior occipital gyrus; and right paracentral lobe to left paracentral lobe. **(B)** In the LTLE group, right fusiform gyrus to left middle temporal gyrus, right medial superior orbital gyrus, left inferior temporal gyrus, left medial superior orbital gyrus and left inferior parietal gyrus; left middle temporal gyrus to right calcarine sulcus; right inferior occipital gyrus to left inferior orbital gyrus; left inferior temporal gyrus to left thalamus; left middle temporal gyrus to right lingual gyrus, right middle occipital gyrus, left thalamus, right middle orbital gyrus, and left paracentral lobe; right middle occipital gyrus to left pallidum, left middle temporo-parieto-occipital junction to right hippocampus; right lingual gyrus to left supramarginal gyrus, left inferior parietal gyrus, left inferior temporal gyrus, and left middle temporo-parieto-occipital junction; right calcarine sulcus to left inferior temporal gyrus; left medial superior orbital gyrus to left lingual gyrus; left middle temporal gyrus to right hippocampus, and right thalamus; left medial superior orbital gyrus to right superior occipital gyrus; and right superior occipital gyrus to left inferior parietal gyrus. **(C)** In the ETLE group, left inferior parietal gyrus to right caudate nucleus; left middle temporal gyrus to right postcentral gyrus and right supramarginal gyrus to right Rolandic area; left inferior orbital gyrus to left medial superior orbital gyrus; right postcentral gyrus to left supramarginal gyrus; left middle temporo-parieto-occipital junction to left superior parietal gyrus; left middle temporal gyrus to right parahippocampal gyrus; right putamen to left middle occipital gyrus; right postcentral lobe to left supramarginal gyrus, left opercular part of inferior frontal gyrus, left dorsal superior frontal gyrus, right inferior occipital gyrus, and right Heschl’s gyrus; left postcentral lobe to left supramarginal gyrus and left putamen; left dorsal superior frontal gyrus to right Heschl’s gyrus, right Rolandic area, and right middle occipital gyrus; right thalamus to right precentral gyrus; right middle temporo-parieto-occipital junction to left middle temporal gyrus and right Rolandic area; right hippocampus to right supramarginal gyrus; left parahippocampal gyrus to left superior parietal gyrus; and right medial superior orbital gyrus to left superior parietal gyrus and left lingual gyrus to right middle occipital gyrus.

All the RTLE, LTLE, and ETLE patients exhibited significant alterations in the cerebral regions compared with HCs (Figures 2A–C). The top 25 brain regions of most significant difference in edges and nodes were visualized (*p <* 0.01, FDR corrected; Figures 3A–C).

### The edge connection signature

In the RTLE group vs. HCs, edge connectivity signatures were significantly distributed between the right Rolandic area and the left superior parietal gyrus, right supplementary motor area, right caudate nucleus, right cerebellar crus 1, right lingual gyrus, left paracentral lobe, left inferior frontal gyrus, triangular part, left Rolandic area, left inferior occipital gyrus, and left cerebellar crus 1. Additional edge connectivity signatures included left superior parietal gyrus to right superior temporal gyrus, right middle temporo-parieto-occipital junction, right hippocampus, right parahippocampal gyrus, and right supramarginal gyrus, along with right cerebellar crus 1 to right precentral gyrus.

In the LTLE group vs. HCs, edge connectivity signatures were significantly distributed in the right fusiform gyrus to the left middle temporal gyrus, the right medial superior orbital gyrus, the left inferior temporal gyrus, the left medial superior orbital gyrus, and the left inferior parietal gyrus. Additional connections included left middle temporal gyrus to right calcarine sulcus, right hippocampus, and right thalamus; left inferior temporal gyrus to left thalamus; and left middle temporal gyrus to right lingual gyrus, right middle occipital gyrus, left thalamus, right middle orbital gyrus, and left paracentral lobe.

In the ETLE group vs. HCs, edge connectivity signatures included left inferior parietal gyrus to right caudate nucleus; left middle temporal gyrus to right postcentral gyrus and right supramarginal gyrus to right Rolandic area; right putamen to left middle occipital gyrus; right postcentral lobe to left supramarginal gyrus, left opercular part of inferior frontal gyrus, left dorsal superior frontal gyrus, right inferior occipital gyrus, and right Heschl’s gyrus; left postcentral lobe to left supramarginal gyrus and left putamen; left dorsal superior frontal gyrus to right Heschl’s gyrus, right Rolandic area, and right middle occipital gyrus; right thalamus to right precentral gyrus; and right hippocampus to right supramarginal gyrus.

### The weighted node connection signature

There was a corresponding similar variation in the connectivity strength of nodes in the network. In the RTLE group, the node signatures in connectivity were dominantly distributed in the bilateral cerebellum, right Rolandic area, left triangular part of inferior frontal gyrus, left superior parietal gyrus, right lingual gyrus, left middle orbital gyrus of frontal lobe, left postcentral gyrus, and right precentral gyrus. In the LTLE group, significant node signatures were observed in the left middle temporal gyrus, right fusiform gyrus, right lingual gyrus, left inferior temporal gyrus, left medial superior orbital gyrus, left inferior parietal gyrus, right middle occipital gyrus, left middle temporo-parieto-occipital junction, right calcarine sulcus, right superior occipital gyrus, left thalamus, and right hippocampus. In the ETLE group, the significant node signatures were observed in the right postcentral gyrus, left middle temporal gyrus, left dorsal superior frontal gyrus, right Rolandic operculum area, left supramarginal gyrus, and left superior parietal gyrus.

### Part 3. The correlation between treatment prognosis and edge connectivity strength of the cerebral region

**Figure 4:**
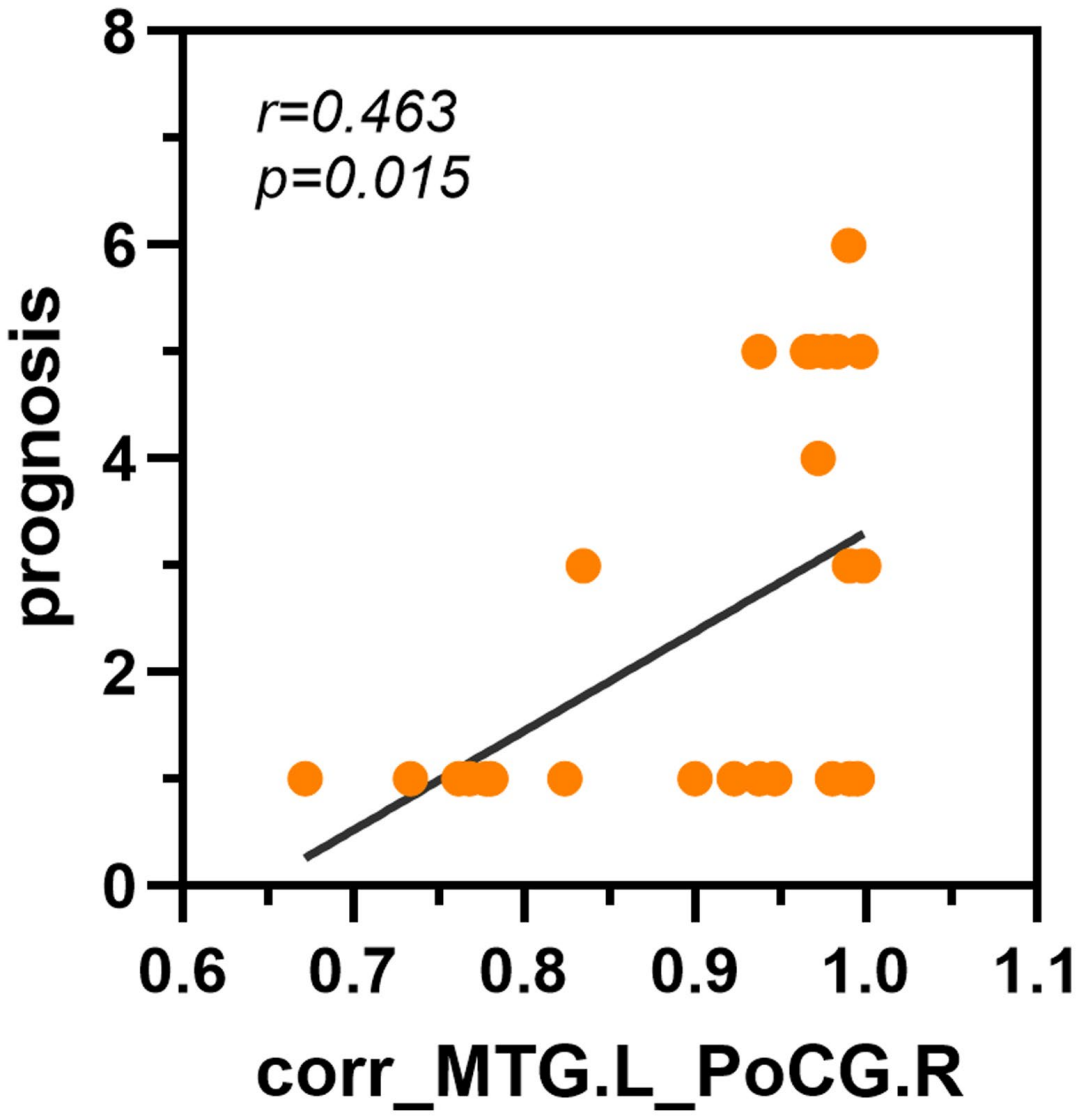
Analysis of correlation between weighted connectivity strength and outcome in the ETLE group (*p* < 0.05). For the outcome scores (*y*-axis), the lowest score (1) corresponds to the best outcome (Engel I) and the highest score to the worst outcome (Engel VI). This study found a positive correlation between the edge-weighted connectivity strength in the left MTG to right PoCG and the outcome (the higher the weighted connectivity strength, the worse the outcome).

In an exploratory analysis of the ETLE group, a positive monotonic association was observed between the strength of specific edge connections and poorer prognosis (*p* < 0.05, Figure 4). Specifically, significant associations were identified in the following edges: left middle temporal gyrus to right postcentral gyrus (*p* = 0.015, *r* = 0.463), right supramarginal gyrus to right Rolandic area (*p* = 0.019, *r* = 0.449), left inferior orbital gyrus to left medial superior orbital gyrus (*p* = 0.03, *r* = 0.418), left middle temporo-parieto-occipital junction to left superior parietal gyrus (*p* = 0.02, *r* = 0.441), right inferior occipital gyrus to right precentral gyrus (*p* = 0.026, *r* = 0.427), left postcentral lobe to left putamen (*p* = 0.019, *r* = 0.448), left parahippocampal gyrus to left superior parietal gyrus (*p* = 0.019, *r* = 0.448), right medial superior orbital gyrus to left superior parietal gyrus (*p* = 0.008, *r* = 0.501), right postcentral gyrus to right Heschl’s gyrus (*p* < 0.001, *r* = 0.652), and right Rolandic area to left dorsal superior frontal gyrus (*p* = 0.003, *r* = 0.552). In addition, in the RTLE and LTLE groups, no significant correlations were found.

## Discussion

There were three major highlights and clinical implications in this study. First, this research conducted an analysis of the changes in the whole individual metabolic network based on the first-order of SUV parameters across the three groups. Three mainstream approaches are commonly adopted in image processing to extract textural features from ROIs, namely: first-order features, second-order features, and higher-order features (22). The first-order features, such as SUV metrics, entropy, energy, maximal correlation coefficient, and low gray-level run emphasis, exhibited small variations. These features were more suitable for reproducible auto segmentation and tumor assessment, and the reason remains that the first-order features do not contain information about the spatial intensity distribution and are more stability and reliability than the second- and higher-order features in PET imaging (22, 23). By analyzing the statistical correlations of cerebral glucose metabolic similarity between pairwise brain regions based on the first-order features, individual metabolic connection patterns may be effectively characterized.

Second, the primary finding of this study revealed key disparities in metabolic network connectivity across cerebral regions among different subtypes when compared with HCs. The ETLE patients showed wider spreading signatures than those with TLE, particularly in the unilateral temporal gyrus, postcentral gyrus, supramarginal gyrus, frontal gyrus, Rolandic area, parietal gyrus, occipital gyrus, precentral gyrus, and putamen. ETLE is more challenging to diagnose and treat surgically due to diffuse epileptogenic activity on scalp EEG, more extensive EZ, and vicinity to eloquent cortex (24). An earlier study showed that ETLE was generally associated with poor surgical outcomes, with estimated seizure freedom ranging from only 27 to 60% (25–28), whereas 65–73% of TLE patients achieved freedom from disabling seizures for at least 1 year postoperatively. This discrepancy may be explained by the observation that increased edge connectivity signatures imply more extensive network activity, which was consistent with Owen TW’s research. Widespread functional abnormalities were related to outcome in a recent ETLE study using the magnetoencephalographic data (29). Collectively, these findings suggest that more widespread structural and functional abnormalities beyond the EZ are associated with poorer post-surgical outcomes (30).

Third, ^18^F-FDG PET parameters were used to evaluate the signature of cerebral network hubs. Notably, this study identified that the right Rolandic area may serve as a key hub region exhibiting subtype-specific connectivity alterations across the RTLE groups, particularly to the cerebellum and contralateral parietal lobe in the results. Fortunately, due to the detailed nature of the relevant clinical data in this research, we may match the brain activity changes with the corresponding improvement in symptoms. This finding is closely associated with common auras and secondary generalized seizures. Previous neuroimaging studies have indicated that the Rolandic area were the original area of oroalimentary automatisms (OAAs) with temporal epilepsy (31, 32), which further confirms that this symptom is more frequently observed in the RTLE group. These findings extend this notion by demonstrating that the involvement of this region is highly dependent on the lateralization and location of the epileptogenic focus. The cerebellum also emerged as an important network signature hub, particularly in the RTLE. Additionally, alterations in the cerebellum involved in the pathogenesis of epilepsy have received increased attention, and this network is recognized as a potential therapeutic target (33). Tang et al. (14) demonstrated distinct topological characteristics of cerebellum in the metabolic networks between MTLE and NTLE, and it was identified as a prognosis factor in MTLE group; Similarly, Oyegbile (34) reported that patients with TLE exhibited significantly reduced total cerebellar volumes compared with HCs, primarily attributed to a reduction in gray matter volume (GMV), the major finding was the variable impact of chronic TLE across cerebellar lobes and tissue types.

The major signature network hub was located between the right fusiform gyrus and left middle temporal gyrus in the LTLE patients, which was consistent with the research by Trimmel et al. (35), who utilized fMRI in 35 LTLE patients, visual naming of elicited activation in left fusiform gyrus might represent an expression of impaired recruitment of language networks caused by an early onset of epilepsy and prolonged disease duration. This is in line with clinical findings showing that impaired naming performance is associated with a longer duration of epilepsy (36, 37). Compared with RTLE patients, stronger correlations were observed in LTLE patients, supporting the reorganization of ipsilateral temporal lobe language networks to the seizure onset zone. Notably, another intriguing finding emerged that the left middle temporal gyrus had signature connectivity to almost all right cerebral regions, such as right fusiform, middle occipital gyrus, hippocampus, orbital part of middle frontal gyrus, and calcarine cortex. The perspective can be interpreted within the framework of the theory proposed by Bettus^,^ research (38), which was the first to demonstrate that decreased basal functional connectivity with epileptogenic networks, accompanied by increased contralateral connectivity, possibly reflects compensatory mechanisms.

Finally, the predominance of cerebral connectivities in ETLE was observed between the unilateral temporal gyrus and the postcentral gyrus, the superior frontal gyrus (medial part), along with the bilateral putamen, the unilateral thalamus, Rolandic operculum gyrus, supramarginal gyrus, and the parietal gyrus. These findings were consistent with the results of Krigel et al., who postulated that the various spread patterns can be explained using the close anatomical connections among the orbital frontal region, mesial temporal structures, and frontal structures (39). The results implied that ETLE epilepsy (11/15) could explore the activity of sensorimotor, language, and memory-related brain regions (postcentral gyrus and Rolandic operculum), while activating emotion-related cerebral areas (amygdala and frontal cortex), which may provide a neural mechanism corresponding to the improvement of clinical symptoms (40).

Focal epilepsy is a type of neurological disorder characterized by disturbance in movement, sensation, emotion, consciousness, and memory. The comorbidity and symptom variability of the disease require subgroup research on specific epilepsy types to explore its pathogenesis. In this research, our novel semiquantitative SUV analysis of cerebral network connectivity offers the advantages for individual classification and prognosis estimation, and represents the first attempt to distinguish RTLE, LTLE, and ETLE. Therefore, differences in the clinic exist in the microscopic metabolic connections leading to distinct network structures and generalized pathways.

The present study had several limitations. First, the sample size is relatively small, and the follow-up time is short. Second, this study adopted a retrospective single-center design, which inherently leads to selection biases. Third, the semiquantified relative ^18^F-FDG SUV parameters are only capable of offering certain non-specific insights into neuropsychiatric disorders, although the cerebellum is widely used as a reference in PET imaging and these data showed no gross cerebellar hypometabolism. Recent evidence suggests it may be involved in epilepsy propagation networks, and subtle network-level changes cannot be entirely excluded, which is a limitation of the SUVR methodology. Future studies may benefit from absolute quantification or data-driven scaling approaches. Finally, the ordinal nature of the outcome scale and the exploratory framework of this study, these findings should be interpreted as preliminary observations. In further research, it may be advantageous to use molecular probes targeting synapses and neuroinflammation, which are associated with the underlying mechanism of focal epilepsy, to enable more direct investigation of the topological attributes of neuronal metabolism.

## Conclusion

To the best of the knowledge, this is the first research to explore the unique distinguishing characteristics of metabolic connectivity topology derived from first-order features of ^18^F-FDG PET, by comparing individuals with RTLE, LTLE, and ETLE to HCs. The relationships and connections among various cerebral regions are highly complex. As a retrospective study, this study performed correlation analyses to examine the associations between changes in metabolic activity, improvements in clinical symptoms, and prognosis. While these findings reveal subtype-specific network signatures, the direct clinical applicability for individual patient diagnosis or prognosis prediction requires prospective validation and should be interpreted with caution, given the retrospective design. This study provides novel methods and insights into the mechanism of ^18^F-FDG PET metabolic connectivity, and further studies with larger sample sizes and longer follow-up are valuable.

## Data Availability

The original contributions presented in the study are included in the article/supplementary material, further inquiries can be directed to the corresponding author.

## Glossary

HS: Hippocampal sclerosis
FCD: Focal cortical dysplasia
TLE: Temporal lobe epilepsy
FLE: Frontal lobe epilepsy
ETLE: Extra temporal lobe epilepsy
EZ: Epileptic zone
^18^F-FDG PET: 2-Deoxy-2-[^18^F]fluorodeoxyglucose positron emission tomography
MRI: Magnetic resonance imaging
fMRI: Functional MRI
HCs: Healthy controls
MTLE: Mesial TLE
NTLE: Neocortical TLE
RTLE: Right TLE
LTLE: Left TLE
ILAE: International League Against Epilepsy
VEEG: Video-electroencephalography
MDT: Multidisciplinary team
AEDs: Antiepileptic medications
T1WI: T1-weighted image
T2WI: T2-weighted image
FLAIR: Fluid-attenuated inversion recovery
DWI: Diffusion-weighted imaging
TR: Repetition time
TE: Echo time
FOV: Field of view
ROI: Region of interest
AI: Asymmetric index
AAL: Anatomical automatic labeling
SUV_mean_: Mean standardized uptake value
SUV_meanstd_: Standardized deviation from the SUV_mean_
SUV_max_: Max standardized uptake value
SUV_maxstd_: Standardized deviation from the SUVmax
SUVR: SUV ratio
IQR: Interquartile range
EEG: Electroencephalography
OAAs: Oroalimentary automatisms
GMV: Gray matter volume.
